# Association of Depression With Cardiovascular Diseases

**DOI:** 10.7759/cureus.26296

**Published:** 2022-06-24

**Authors:** Zain I Warriach, Sruti Patel, Fatima Khan, Gerardo F Ferrer

**Affiliations:** 1 Family Medicine, Larkin Community Hospital, Miami, USA; 2 Research and Academic Affairs, Larkin Community Hospital, Miami, USA; 3 Psychiatry, Larkin Community Hospital, Miami, USA

**Keywords:** depressive disorder, mortality, morbidity, cardiovascular disease, depression

## Abstract

Depression has long been associated with cardiovascular morbidity and mortality. We have reviewed the various factors (hormonal, inflammatory, neuroimmune, and behavioral) involved in depression and associated cardiovascular risk factors. Elevation of glucocorticoids due to activation of the hypothalamic-pituitary-adrenal (HPA) axis in chronic stress of depression results in hyperglycemia, causing insulin resistance, which is a risk factor for heart diseases. This increase in glucocorticoids also stimulates the production of pro-inflammatory cytokines interleukin (IL)-1, IL-6, and tumor necrosis factor-alpha. Literature also showed that chronic stress in depression activates platelet receptors resulting in endothelial dysfunction and cardiovascular morbidity. It has been shown by various studies that depressed patients are more prone to unhealthy lifestyles like eating more processed food, physical inactivity, smoking, and alcohol consumption resulting in weight gain and insulin resistance. Further in the literature, we reviewed some genetic factors associated with depression and cardiovascular outcomes. Elevated glucocorticoids reduce brain-derived neurotrophic factor-dependent upregulation of glutamate receptors involved in various neural circuits associated with depression and neural diseases by suppressing microRNA-132 expression. In depressed obese patients, proprotein convertase subtilisin/kexin type 9 (PCSK-9), a regulator of low-density lipoprotein cholesterol, has been shown to be associated with insulin resistance. This review sheds light on the importance of diagnostic, preventive, and treatment strategies in depressed patients to reduce overall cardiovascular morbidity and mortality.

## Introduction and background

Depression, one of the serious mental health illnesses, has been shown in the literature to have a bidirectional relationship with cardiovascular disease (CVD) morbidity and mortality [1-7]. We reviewed various factors (inflammatory, hormonal, genetics, neuroimmune, and behavioral) involved in the pathophysiology of depression and associated cardiovascular outcomes. The increased stress in depression activates the hypothalamic-pituitary-adrenal (HPA) axis, resulting in the elevation of the glucocorticoids and hyperglycemia, causing insulin resistance, a proven risk factor for diabetes associated with cardiovascular outcomes [8-10]. Corticotropin-releasing factor (CRF) regulates the HPA axis and neuroimmune system in depression [11-14]. Elevated CRF levels stimulate the production of pro-inflammatory cytokines interleukin (IL)-1, IL-6, and tumor necrosis factor (TNF)-alpha and decrease anti-inflammatory cytokine IL-10 [15-18]. Further, the literature showed that chronic stress in depression activates platelet receptors, signaling, and degranulation resulting in cardiovascular outcomes [19,20]. Platelet distribution width (PDW) is a potential biomarker of depression involved in depression and CVDs. So markers of platelet activation may reflect the stage of depression and have a prognostic and intervention role in its treatment. We had also shown the role of behavioral and lifestyle factors in depression and CVDs. Depressed patients are usually inclined to unhealthy lifestyles with less physical activity, processed diet, weight gain, and smoking leading to obesity and insulin resistance. Several studies showed the dietary inflammatory index (DII) score is associated with depression, anxiety, atherosclerosis, and stroke outcomes. The data showed an increase in the incidence of CVDs with the pro-inflammatory diet versus the anti-inflammatory diet. Literature showed that a diet rich in fruit and vegetables, moderate alcohol consumption, no smoking, and physical activity improves the cardiovascular outcome. Further in the literature, we showed the role of genetic factors in the association of depression with CVDs. Elevated glucocorticoids reduce brain-derived neurotrophic factor (BDNF)-dependent upregulation of glutamate receptors by suppressing microRNA-132 expression, which is involved in various neural circuits and associated with the pathophysiology of mental disorders and neural diseases. Another study showed that elevated levels of proprotein convertase subtilisin/kexin type 9 (PCSK9), a mediator of low-density lipoprotein (LDL) cholesterol, are associated with insulin resistance in depressed obese subjects. Therefore, PCSK9 could play a role as a potential biomarker in depressed obese patients to identify people who are at increased risk of CVD.

Methods

In this study, we conducted focused literature searches using databases such as PubMed and Google Scholar to identify relevant original research and review articles. Search terms focused on epidemiology (e.g. “cardiovascular mortality” and “cardiovascular morbidity”), associations between depression or depressive disorder and CVD (e.g. “depression increases cardiovascular disease”), and health behavior mechanisms (e.g. “depression inflammation” and “stress”). Titles and abstracts from these searches were reviewed, full-text articles were obtained for relevant manuscripts, and reference lists were reviewed to identify additional manuscripts appropriate for review. This is a traditional review and not a systematic review, so no PRISMA (Preferred Reporting Items for Systematic Reviews and Meta-Analyses) or AMSTAR (Assessment of Multiple Systematic Reviews) guidelines are used.

## Review

Depression, one of the debilitating mental illnesses, has long been associated with CVD morbidity and mortality [1-3]. A bidirectional role between the two has been proven in the literature [4-7]. In this traditional review, we elaborate on the possible known molecular mechanisms involved in the pathophysiology of depression, which directly and indirectly facilitate the mechanisms involved in CVD pathology. The review also shows that chronic stress in depression could lead to endothelial dysfunction, platelet activation, and signaling leading to CVD outcomes. There are inflammatory, hormonal, genetic, behavioral, and neuroimmune-related mechanisms involved in the pathophysiology of depression and CVD.

HPA axis changes in depression and CVD

The unusual stress in depression activates the HPA axis by increasing the concentration of glucocorticoids [8-10]. The increase in the concentration of glucocorticoids causes hyperglycemia, resulting in insulin resistance, which ultimately leads to diabetes mellitus with cardiovascular consequences. CRF is a hypothalamic hormone that regulates the activity of the HPA axis and the neuroimmune system in depression. This hormone also exerts its effects on other organs such as the skin, gastrointestinal system, and cardiovascular system [11-14]. Elevated cortisol concentrations in salivary and plasma cortisol levels are greater in individuals suffering from depression [13]. Additionally, elevated CRF levels due to stress in depression stimulate the production of pro-inflammatory cytokines IL-1, IL-6, and TNF-alpha by peripheral immune cells. These peripheral cytokines can cross the blood-brain barrier and activate astrocytes and microglia in the central nervous system. These activated cells can further secrete more pro-inflammatory cytokines causing neuroinflammation and producing depression-like behavioral symptoms. Inflammatory cytokines are abnormally increased in depressive disorder [13]. On the other hand, anti-inflammatory cytokines like IL-10 play a different role in the CRF-driven regulation of depression. IL-10 produced in the pituitary and hypothalamus limits the immune responses by inhibiting the production of pro-inflammatory cytokines.

One of the clinical studies showed an increase in the production of IL-10 after treatment with antidepressants. Also, IL-10 stimulates the secretion of adrenocorticotropic hormone (ACTH), which inhibits the secretion of CRF by the negative feedback mechanism of the HPA axis. CRF stimulates the secretion of pro-inflammatory cytokines and suppresses the secretion of anti-inflammatory cytokines. This interaction between CRF and cytokines could play a crucial role in finding an effective therapeutic strategy for depression by targeting the network of cytokines and CRF in the central nervous system. Patients with depression reported elevated levels of inflammatory markers IL-1, IL-6, and TNF-alpha, which play a major role in the development of atherosclerosis and coronary artery disease [15-18]. Platelet activation through specific receptors, signaling pathways, and degranulation results in tissue inflammation, which can initiate and aggravate thrombotic complications resulting in cardiovascular morbidity [19,20].

PDW, a potential novel biomarker for depression, has been associated with both depression and other neurological abnormalities like Alzheimer’s disease and Parkinson’s disease. So markers of platelet activation may reflect the stage of mental disease progress and are a promising biomarker to investigate the major depressive disorder and its comorbidities in the future. One study showed that depressive symptoms leading to weight gain activate two distinct inflammatory pathways: the release of IL-6 from adipose tissue and leptin-induced upregulation of IL-6 release by white blood cells. In another study, in patients with the earlier acute coronary syndrome, the relation of depressive symptoms to C-reactive protein and pathogen burden (cytomegalovirus, Epstein-Barr virus, and herpes simplex virus) is assessed through self-report and observer ratings [21]. The levels of inflammatory markers (IL-6, C-reactive protein, and TNF-alpha) are determined along with antibody titers to three latent viruses associated with atherosclerosis [22-26]. Patients with more severe depressive symptoms reported higher levels of C-reactive protein and higher levels of seropositivity to latent viruses.

Lifestyle in depression and CVD

Further, we discussed the lifestyle and behavioral factors in the association of depression with CVD. Patients with depression usually carry an unhealthy lifestyle such as smoking, alcohol use, unhealthy diet, poor medication compliance, and physical inactivity, leading to weight gain, obesity, and poor health outcomes [27-29]. These patients are also less motivated to change their lifestyles and adopt healthy behaviors. In one of the cross-sectional studies, it has been shown that symptoms of depression are associated with physical inactivity and not changed by the presence of CVD or gender difference. Future research should aim at lifestyle interventions in depressed patients by increasing physical activity and simultaneously improving symptoms of depression, despite the difference in gender and the presence of CVD.

It has been shown in one of the studies on an aging population over 80+ years that no smoking, moderate alcohol consumption, healthy diet, physical activity, and normal weight are prognostic health parameters in the aging population. In this study, the severity of depressive symptoms is directly associated with worse cardiovascular outcomes. Current evidence suggests that women with depression experience a higher risk of coronary artery disease than men, and females with coronary artery disease have more chances of having depression than men. Regardless of the similar outcomes of CVD with the risk factors like hypertension, weight gain, and increase in cholesterol between men and women, prolonged smoking is significantly more deleterious for women than men [30-32].

Further in the literature, we have shown the importance of dietary inflammation and severe mental illness like depression. Higher calorie diets like energy drinks, saturated fat, and simple carbohydrates tend to increase peripheral inflammatory markers while a diet rich in fruits, vegetables, and fiber reduces inflammation. This evidence has been taken from various cross-sectional, observational, and experimental findings [33-36]. For example, after a meal of processed carbohydrates and energy drinks, there will be hyperglycemia and hyperinsulinemia, which may promote inflammation by increasing the production of free radicals and pro-inflammatory cytokines.

In the Asklepios study, an association between DII and inflammatory markers was found. The DII was calculated using the food frequency questionnaire (FFQ)-derived dietary information and compared against inflammatory markers like IL-6, C-reactive protein, homocysteine, and fibrinogen. Multivariable analysis after adjusting for different variables had shown significant positive associations between DII and the inflammatory markers (IL-6 and homocysteine). These results had proven the fact that diet has an important role in modifying inflammation [37]. To further consolidate this association between DII and depression, we reviewed another study conducted in Iran, which studied depression and anxiety in the stroke and heart atherosclerotic disorder (MASHAD) study population [33,34]. To explain this hypothesis, we recall the role of the HPA axis and glucocorticoids. The increased stress in depression leads to elevated glucocorticoid and glucose levels through gluconeogenesis, causing obesity and insulin resistance. The DII was calculated in the middle-aged Iranian population of both men and women to determine the possible inflammatory effect of the diet. The data gathering of this DII score is based on the reported consumption of up to 45 food parameters; 28 out of the 45 food parameters derived from the FFQ were used. DII score ranges from most anti-inflammatory to most pro-inflammatory. They also categorized the score into quartiles, defined as Q1, Q2, Q3, and Q4. Q1 is labeled as the most anti-inflammatory and Q4 is the most pro-inflammatory. After adjusting for confounding variables in this study, in women, the Q3 and Q4 quartiles of the DII score were associated with an increased risk of depression compared to the Q1 (also the reference group) of the DII score. They concluded that there was a significant association between DII score and severe depression among women but not in men of this Iranian study population. Therefore, adaptive measures for an anti-inflammatory diet could be an effective intervention and prevention for depressive symptoms. Further research should focus on the DII score to predict CVD outcomes such as atherosclerosis and other indicators of heart disease like intimal thickening, plaque formation, and cardiac output [38].

Genetics in depression and CVD

There are genetic factors associated with the development of depression and the cardiovascular outcome in the patient. A study shows the interaction of steroid hormones and neurotrophic BDNF [39]. Stress-induced elevation in glucocorticoids reduces the BDNF levels and neuronal functions associated with it. Alteration in the level of BDNF in the CNS is involved in the pathophysiology of numerous brain diseases such as Parkinson’s disease, Alzheimer's disease, cerebrovascular accident (CVA), and mental disorders. Currently, in the literature, we found that glucocorticoids reduced BDNF-dependent upregulation of glutamate receptors by suppressing microRNA-132 expression [40,41].

**Figure 1:**
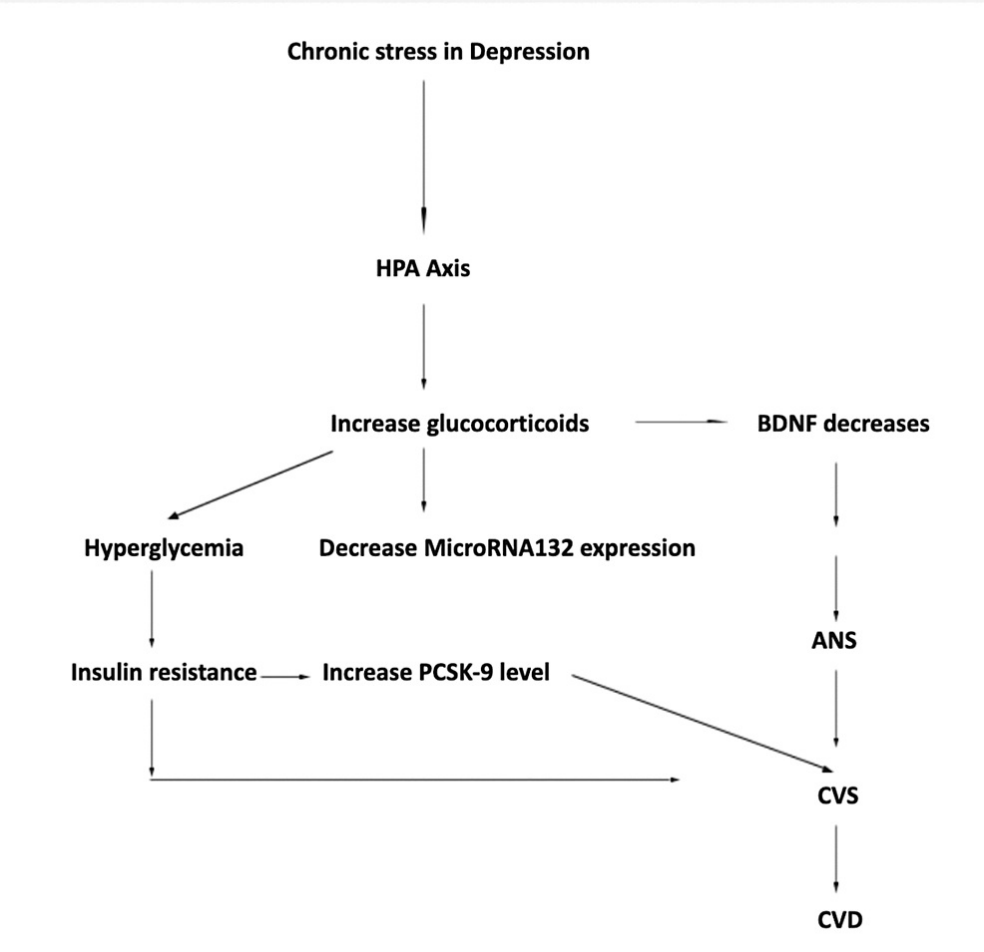
An integrative model showing the bidirectional association between depression and CVD. CVD, cardiovascular disease; HPA, hypothalamic-pituitary-adrenal; BDNF, brain-derived neurotrophic factor; PCSK-9, proprotein convertase subtilisin/kexin type 9; ANS, autonomic nervous system; CVS, cardiovascular system.

Other steroid hormones like estrogen are also involved in numerous neuronal events including cell survival and synaptic plasticity, and regulation of BDNF levels. The role of estrogen is well documented in the pathogenesis of Alzheimer’s disease, Parkinson’s disease, and mental illnesses. All individuals have their own degree of neuroplasticity, which is the ability of the neural circuits in the brain to reform and reorganize in response to the new learning experiences or after injury due to their unique experiences. This may also play a role in the treatment response of different antidepressants. As the central nervous system activity is coordinated with various internal organ functions, neuroplasticity may be associated with other diseases. Depression, a disorder of disorderly neuroplasticity, is associated with cardiovascular morbidity and mortality. MicroRNA-132 has a role in neuroplasticity and may play a role in the coexistence of depression and cardiovascular function. Figure 1 shows the bidirectional association of depression with CVD.

Depression is two times more prevalent in people with diabetes and is associated with a 60% increase in the incidence of type 2 diabetes, an independent cardiovascular risk factor. Several studies have shown that some anti-hyperglycemic agents like insulin, metformin, glyburide, pioglitazone, and others reduce depressive symptoms in the presence or absence of diabetes. This antidepressant activity of these anti-hyperglycemic agents is mediated by decreasing blood glucose levels, reducing central oxidative stress and inflammation, and regulating the HPA axis [42].

PCSK9, a mediator of LDL cholesterol, has been associated with a large number of cardiovascular risk factors, including insulin resistance. A study investigated the association of depression with insulin resistance, especially in obese patients and PCSK9 levels, and the cardiovascular outcome. The study results showed an increase in the PCSK9 levels in the obese subjects, associated with the Beck Depression Inventory (BDI). As depression is associated with an increased risk of diabetes, the identification of PCSK9 levels in depressed obese patients may identify people who are at increased risk of cardiovascular morbidity and could benefit the most from targeted intervention [43]. Table 1 demonstrates five important studies that focus on the associations between CVD and depression.

**Table 1:** Relevant clinical trials and literature that explored the association between CVD and depression. CVD, cardiovascular disease; COMT, catechol-O-methyltransferase; KNHANES, Korea National Health and Nutrition Examination Survey; MSD, moderate to severe depression; DII, dietary inflammatory index.

Authors	Year	Methodology	Outcomes/results
Park et al. [7]	2020	Retrospective cohort study	Depression increased the risk of ischemic heart disease by 38% and cerebrovascular disease by 46% among older adults in Korea.
Almas et al. [44]	2018	Longitudinal cohort study	High COMT activity increased the risk of CVD in depressed patients, higher in women compared to men (OR: 7.0; 95% CI: 3.0-14.0 versus OR: 2.1; 95% CI: 1.0-6.8).
Ghazizadeh et al. [33]	2020	Cross-sectional study	Association between DII score and depression among women but not men. Of patients, 37.1% (n = 2631) were found to have mild to severe depression, and 50.5% (n = 3580) were affected by mild to severe anxiety.
Song et al. [10]	2019	KNHANES data analysis	The mean CVD risk of the MSD group was higher than that of the normal group (p < 0.05). This study also showed how CVD risk increases as the depression worsened (p < 0.01).
Melin et al. [31]	2019	Cross-sectional study	Low high-density lipoprotein cholesterol levels are associated with increasing triglyceride levels (p < 0.001), increasing high-sensitive C-reactive protein (hs-CRP) levels (p = 0.021), younger age (p < 0.001), male sex (p < 0.001), and depression (p = 0.045).

In one of the cohort studies done among the Swedish population, the role of catechol-O-methyltransferase (COMT Val158Met) was discussed in depressed patients and the future risk of CVD. COMT enzyme is involved with the breakdown of dopamine, estrogen, and other catecholamines, which have a role in the pathophysiology of depression. This study showed that varying degrees of COMT enzymatic activity influence CVD outcomes in depressed patients. COMT valine allele has more enzymatic activity versus the methionine allele. So the higher enzymatic activity of the COMT Val158Met (substitution of methionine for valine at codon 158 encoded by a single nucleotide polymorphism) is associated with the greater degradation of dopamine and increased risk of depression and CVD [44].

## Conclusions

This article reviewed the available literature to investigate the various associations between depression and CVD. We deduced that various hormonal, neuroimmune, behavioral, and genetic factors are associated with depression and cardiovascular morbidity and mortality. Chronic stress due to depression causes hyperglycemia by activation of the HPA axis resulting in insulin resistance. HPA axis is regulated by CRF, which leads to the secretion of pro-inflammatory cytokines and suppresses the secretion of anti-inflammatory cytokines, eventually leading to heart disease. Lifestyle and behavioral factors such as smoking, alcohol use, unhealthy diet, and physical inactivity also lead to poor health outcomes. Future research should aim at lifestyle interventions in depressed patients by increasing physical activity and simultaneously improving symptoms of depression. Genetic factors also play a major role in depression, such as increased steroid hormones leading to suppression of BDNF and microRNA-132 gene affecting neuroplasticity. Other potential genetic biomarkers such as PCSK-9 and COMT enzyme activity can contribute to this relationship.

It is essential to adopt measures and interventions to diagnose and treat depression and cardiovascular risk factors and to ameliorate cardiovascular morbidity and mortality. Further research should be conducted to diagnose and treat depressed patients in the early stages at risk of CVD to reduce the morbidity and mortality outcomes. Future research studies should focus on prevention strategies for depressed patients at risk of CVD.
